# Supplementary material for: Interaction of Proteins with a Planar Poly(acrylic acid) Brush: Analysis by Quartz Crystal Microbalance with Dissipation Monitoring (QCM-D)
Source: Polymers (Basel). 2020 Dec 30;13(1):122. doi: 10.3390/polym13010122 (PMC7795234; doi:10.3390/polym13010122)
Supplement: Supplementary file 1 [file polymers-13-00122-s001.pdf]

SUPPORTING INFORMATION

Interaction of Proteins with Poly(acrylic acid) Brush: Analysis by Quartz Crystal Microbalance with Dissipation Monitoring (QCM-D)

Jacek Walkowiak [1], Michael Gradzielski [2], Stefan Zauscher [3,*] and Matthias Ballauff [4,*]

[1] Aachen-Maastricht Institute for Biobased Materials (AMIBM), Maastricht University, Brightlands Chemelot Campus, Urmonderbaan 22, 6167 RD Geleen, The Netherlands; j.walkowiak@maastrichtuniversity.nl
[2] Stranski Laboratorium für Physikalische Chemie und Theoretische Chemie, Institut für Chemie, Straße des 17. Juni 124, Sekr. TC7, Technische Universität Berlin, 10623 Berlin, Germany; michael.gradzielski@tu-berlin.de
[3] Mechanical Engineering and Material Sciences, Duke University, Durham, NC 27708, USA
[4] Institut für Chemie und Biochemie, Freie Universität Berlin, Takustraße 3, 14195 Berlin, Germany
* Correspondence: zauscher@duke.edu (S.Z.); mballauff@zedat.fu-berlin.de (M.B.)

I- and pH cycle upon HSA adsorption:

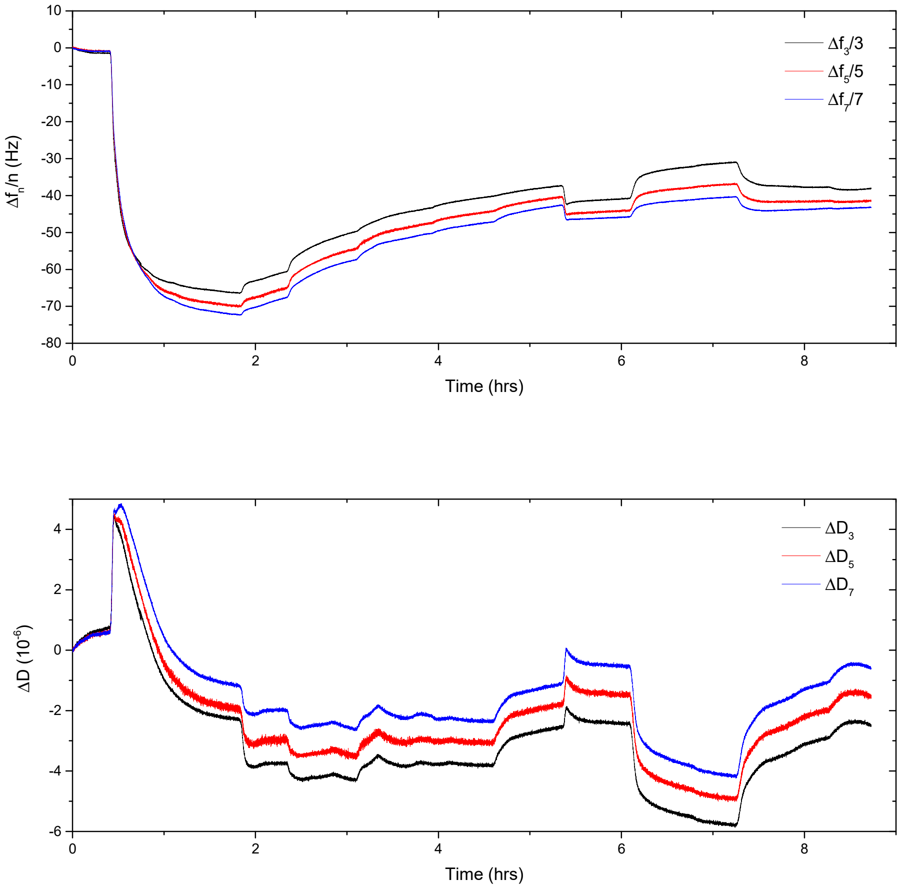

Figure S-1. I- and pH induced response of protein pre-complexed PAA brush monitored by QCM-D. Top panel: QCM-D normalized frequency signal. Lower panel: QCM-D dissipation signal. Results for the third, the fifth, and the seventh overtone are displayed.

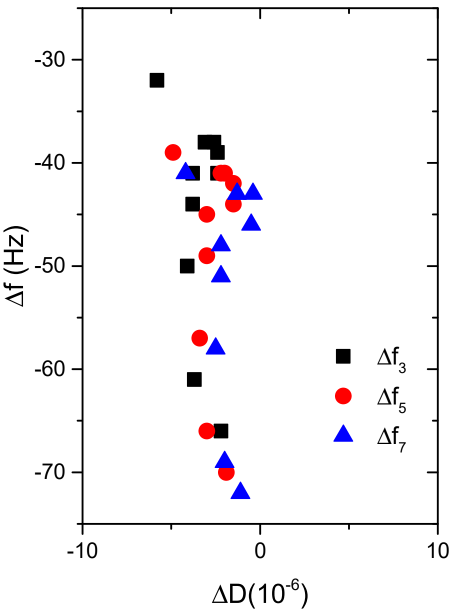

Figure S-2. Distribution of Δf as a function of the corresponding ΔD. Results for the third, the fifth, and the seventh overtone are displayed.

pH induced swelling/deswelling of a protein-free PAA brush:

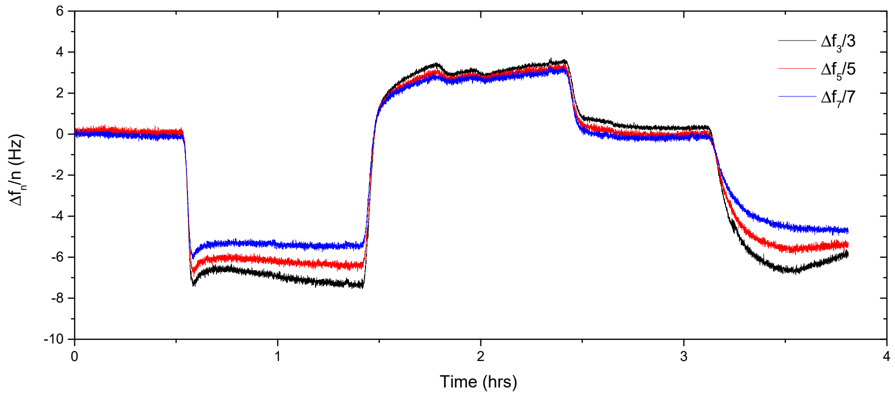

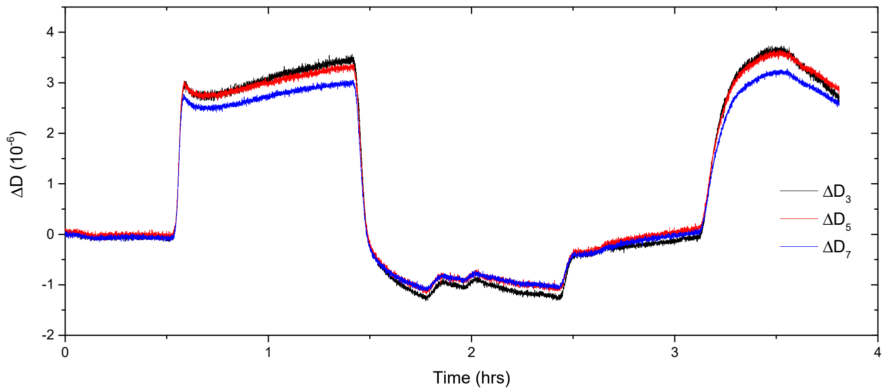

Figure S-3. pH induced response of protein-free PAA brush monitored by QCM-D. Top panel: QCM-D normalized frequency signal. Lower panel: QCM-D dissipation signal. Results for the third, the fifth, and the seventh overtone are displayed.

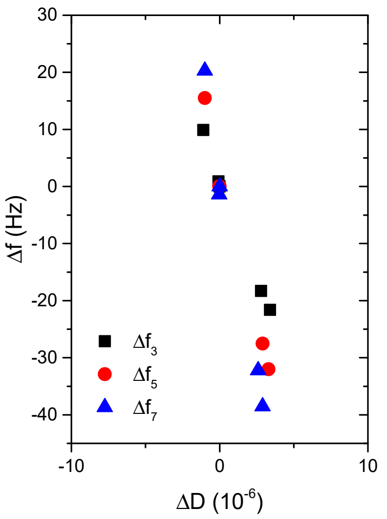

Figure S-4. Distribution of Δf as a function of the corresponding ΔD. Results for the third, the fifth, and the seventh overtone are displayed.

Determination of the number of HSA molecules per PAA chain.

For an arbitrary area of $S = 20\ nm$ with grafting density of $\sigma = 0.35 \pm 0.13\ nm^{-2}$ we have approximately $N_c = 7 \pm 3$ PAA chains.

Mass density at Step II of ionic strength cycle is $353\ Da/Å^2$ (see Table 3), which for an arbitrary area S give $706000\ Da$. By taking into account the molecular weight of a single HSA molecule ($M_{w,\ HSA} = 66.5\ kDa$) we get $N_p = 11$ HSA molecules per area S. Keeping in mind that area S is occupied by approximately 7 PAA chains brings us to the number of HSA molecules per PAA chain ($N_{p/c}$) which in this case is 1.5. It means that approximately three HSA molecules are adsorb per two PAA chains.

In the same fashion we can evaluate that at Step X of the pH cycle $N_{p/c} = 1$, meaning that one HSA molecule is adsorb per one PAA chain.
